# Prevalence and predictors of non-adherence to short-term antibiotics: A population-based survey

**DOI:** 10.1371/journal.pone.0268285

**Published:** 2022-05-19

**Authors:** Basima A. Almomani, Bushra M. Hijazi, Oriana Awwad, Rawand A. Khasawneh

**Affiliations:** 1 Department of Clinical Pharmacy, Faculty of Pharmacy, Jordan University of Science and Technology, Irbid, Jordan; 2 Department of Biopharmaceutics and Clinical Pharmacy, School of Pharmacy, the University of Jordan, Amman, Jordan; University of Turin, ITALY

## Abstract

Non-adherence to antibiotics is a well-known, core player to antibiotic resistance. The current adherence behavior toward short-term antibiotic regimens has never been investigated before in Jordan. This study assessed the prevalence and predictors of non-adherence to short-term antibiotics among Jordanians and investigated participants’ views about different reasons related to antibiotics non-adherence. A cross-sectional face-to-face survey-based interview was conducted in three hospital pharmacies in Jordan. Adults and mother of children (≤12 years old) who completed their short course treatment (<30 day) of oral antibiotic within the last month were recruited. A total of 2000 participants (adults: 1000 and mothers of children: 1000) were included in the study with a response rate of 91.60%. The prevalence of non-adherence was estimated to be 32.10%. Non-adherent respondents scored a lower Medication Adherence Report Scale [16.76±5.02 vs. 23.04 ±3.24] than adherent respondents (p<0.001). Adults without comorbidity and children with higher number of doses per regimen were significantly less adherent to antibiotic [(OR = 0.615, 95%CI = 0.444–0.853, p = 0.004) and (OR = 0.965, 95%CI = 0.950–0.981, p<0.001)], respectively. Patients-related factors were the most common antibiotic non-adherence reason reported by the participants. The multivariate analysis for all the participants (adults and children), indicated that mothers were 2.6 times more likely to be adherent in giving antibiotics to their children than adults (p<0.001). These findings highlight that more than half of the participants were adherent to short-term antibiotics. However, improving the current prescription-related practices and implementing pharmaceutical consultation services upon antibiotic dispensing are encouraged.

## Introduction

Medication adherence is defined as the degree to which the behavior of patient taking medication is consistent with the standard recommendations from a healthcare provider [1]. In clinical practice, non-adherence to prescribed regimens has always been a concern with higher adherence rates usually observed in acute conditions compared to chronic ones [1]. Non-adherence to antibiotics increases the risk of antibiotic resistance with a resultant impact on treatment outcomes and consequently more utilization of healthcare resources [2, 3]. Moreover, leftover antibiotics increase the likelihood of self-medication and potential misuse [4]. A previous study in France reported that only 50% of hospitalized patients were adherent to their antibiotics after hospital discharge [5].

Multiple determinants were reported to affect non-adherence [6]. The ability of healthcare professionals to identify non-adherent subjects is difficult [1]. Although objective methods (e.g. prescription refill, pill counts) are more reliable to measure medicine adherence compared to subjective methods (e.g., patient self-report, questionnaire), they are more expensive and time consuming [2]. Subjective methods on the other hand, are considered simple, practical and useful in clinical settings [1].

A vast body of literature demonstrated lack of adherence to short term antibiotic [4, 5, 7, 8]. However, no studies are available in the middle eastern region regarding this issue. In Jordan, this has never been investigated before. The magnitude of the issue and the factors affecting adherence to short term antibiotic might be different between countries, such as economic factors, culture, healthcare system, socioeconomic status, health literacy, etc… For instance, in Jordan, there is a non-prescription access to antibiotics since they can be purchased over-the-counter from pharmacies, which contributes to their inappropriate use. Recently, Jordanian regulations prohibiting such practice have been issued, yet they are not fully enforced. Previous studies in Jordan have focused on assessing medication adherence in patients with chronic diseases [9–12]. It is also important to assess the Jordanians current adherence behavior toward short-term antibiotic regimens, to identify the distinct characteristics that affect adherence to tailor future interventions accordingly. The main goal of this study was to assess the prevalence and predictors of non-adherence to short-term antibiotics among the Jordanian population. A secondary objective was to investigate participants’ views about different reasons related to non-adherence of antibiotics.

## Materials and methods

### Ethics statement

The ethical approval for this research was obtained by the institutional review boards at the Jordan University of Science and Technology (15/126/2019) and the Ministry of Health (178/2019). Written informed consent was obtained from all the adult participants and from the mothers of children for the minor participants.

### Study setting

A multi-centered, cross-sectional survey-based study was conducted in Jordan from Aug 2019 to March 2020. A convenience sampling method was adopted where eligible subjects were approached at three public referral teaching hospitals across the main geographical areas of Jordan: north (King Abdullah University Hospital), middle (Al-Bashir Hospital), and south (Al-Karak Governmental Hospital). The sampling plan was to recruit equal number of both adults (n = 1000) and mother of children (n = 1000). The research assistant (clinical pharmacists) approached the patients in the waiting area of the outpatients’ hospital pharmacy, explained them the objectives of the study and invited them to participate. Patients were eligible for inclusion in the study if they were adults or mothers of children (≤12 years old) who used a short course treatment (< 30 day) of oral antibiotic within the last month. Those patients who were immunocompromised or used antibiotics for prophylactic indications were excluded from the study. The patients were approached till the target number of the sample was recruited.

After obtaining a written informed consent, a face-to-face interview was conducted by research assistants (n = 3). The research assistants were trained on how to collect data before starting the study to ensure consistency in the data collection. Frequent meetings were arranged by the research team members to confirm effective communication between the research assistants and the patients and the delivering of questions in a simple and understandable way. The time needed for the interview was approximately 15 min.

### Questionnaire development

During the questionnaire preparation, questions related to short-term adherence were selected from the literature [2, 7, 13, 14]. The questionnaire was composed of three parts. The first one included socio-demographic characteristics of the participants such as age, gender, education, employment, family income, source of payment for antibiotic and presence of comorbid disease. The second part included information related to antibiotic use and adherence, such as how many antibiotics were used in the last month, symptoms for using antibiotic, duration/dosing /times interval, and missed days/doses. In the current study, the level of adherence was measured subjectively, and the participants were labeled as non-adherent if they failed to follow the duration and/ or number of daily doses as recommended by healthcare professionals. These measurements were used based on previous studies [2, 14]. The assessment of adherence was also supported by using the Medication Adherence Report Scale (MARS) [15]. MARS has initially developed to measure adherence to long-term treatment, however it has been also used to measure adherence to short term antibiotic use [14]. MARS has been validated among Jordanian’s adults and children [16]. MARS is a continuous scale of five items (“I forget to take them”, “I alter the dose”, “I stop taking them for a while”, “I decide to miss out a dose”, and “I take less than instructed”) with each item being scaled from 1 to 5 leading to a maximum score of 25, which indicates a highly adherent behavior [15]. The third part of the questionnaire included different reasons related to antibiotics non-adherence (patients, therapy, healthcare, and condition) as proposed by the World Health Organization (WHO) [2, 17]. Face validity was performed, and the questionnaire was pre-tested for easy understanding and for clarity through a pilot study (n = 15). The items in the questionnaire were formatted as closed-ended questions. The questionnaire was originally written in English and then translated to Arabic language.

### Statistical analysis

Following data collection, the responses were coded and entered into SPSS (version 20). Descriptive statistics were used to summarize the data for the total sample as counts (percentage) for categorical data and as mean ± standard deviation (SD) for continuous data. Univariate analysis was performed using a Chi square (χ^2^) (categorical variables) and t-test analysis (continuous variables) as appropriate. A multivariate analysis was conducted to determine predictors of non-adherence, using binary logistic regression. A cutoff point of p<0.25 was used for the univariate analysis to determine which variables will be entered in the multivariate analysis. Variables that were investigated as predictors for non-adherence included age, gender, education, employment, monthly income, comorbid condition, source of payment for antibiotic, number of antibiotics used in previous month, and number of doses per regimen. Odds ratio (OR) values and their 95% confidence intervals (95% CI) were calculated. All reported P values were 2-sided with a 0.05 significance level.

## Results

### Demographics

Out of the 4674 patients approached, 2490 were excluded from the study due to different reasons [did not use antibiotic in last month (n = 1872), immunocompromised (n = 230), or used antibiotic for prophylaxis (n = 388)]. Of the 2184 eligible patients for inclusion into the study, 184 refused to participate leaving a total of 2000 (1000 adults and 1000 mothers of children) to be included in the final analysis with a response rate of 91.60%. Among adult participants, the average age was 36 years old and 67.60% of them were females. Approximately 60.00% of the adults had a university degree and 30.30% had a comorbid condition. In contrast, the average age of children was 5 years and 55.30% of them were males. A third of children’ mothers had a university degree and 27.40% of them were employed. Two thirds (66.70%) of children’ mothers had a low monthly family income of less than 500 Jordanian Dinar (JD, 1 JD = 1.41 Dollar) and 12.60% of children had a comorbid condition. Most participants in both groups used antibiotic for one time within the previous month (74.70% for children vs. 70.00% for adults) with respiratory symptoms being the most common cause for using antibiotics (92.40% for children vs. 71.00% for adults). The average number of doses per regimen both groups was 15 (13.98 for children vs. 16.49 for adults). Detailed description of demographic and clinical data is presented in Table 1.

**Table 1:** Demographic and clinical data.

Characteristics^a^	All participants (n = 2000)	Adults (n = 1000)	Children (n = 1000)
Age (years)^b^	20.51 ± 18.34	35.82 ± 13.89	5.20 ± 3.28
Gender Female Male	1123 (56.15) 877 (43.85)	676 (67.60) 324 (32.40)	447 (44.70) 553 (55.30)
Education level^c^ School education^d^ University education	1015 (50.75) 985 (49.25)	394 (39.40) 606 (60.60)	621 (62.10) 379 (37.90)
Geographical area North Middle South	784 (39.20) 979 (48.95) 237 (11.85)	355 (35.50) 434 (43.40) 211 (21.10)	429 (42.90) 545 (54.50) 26 (2.60)
Employment^c^ Unemployed Employed	1357 (67.85) 643 (32.15)	631 (63.10) 369 (36.90)	726 (72.60) 274 (27.40)
Family income^c^ <500JD ≥500JD	1196 (59.80) 804 (40.20)	529 (52.90) 471 (47.10)	667 (66.70) 333 (33.30)
Source of payment for antibiotic^c^ Out of pocket Insurance Both	453 (22.65) 627 (31.35) 920 (46.00)	266 (26.60) 291 (29.10) 443 (44.30)	187 (18.70) 336 (33.60) 477 (47.70)
Presence of comorbidity No Yes	1571 (78.55) 429 (21.45)	697 (69.70) 303 (30.30)	874 (87.40) 126 (12.60)
Causes of using antibiotics^e^ Respiratory symptoms Urinary tract symptoms Gastrointestinal symptoms Others	1634 (81.70) 171 (8.55) 260 (13.00) 366 (18.30)	710 (71.00) 133 (13.30) 132 (13.20) 319 (31.90)	924 (92.40) 38 (3.80) 128 (12.80) 47 (4.70)
Number of antibiotics used in previous month One time ≥ two times	1447 (72.35) 553 (27.65)	700 (70.00) 300 (30.00)	747 (74.70) 253 (25.30)
Number of doses per regimen^b^	15.24 ± 10.27	16.49 ± 11.15	13.98 ± 9.15

^a^ All data expressed as n (%) of patients unless otherwise indicated.

^b^ Data described as mean ± standard deviation

^c^ For mothers of children

^d^ Primary and/or secondary school

^e^ Some participants reported more than one cause

### Prevalence of non-adherence to short-term antibiotics

The prevalence of non-adherence among all the participants (adults and children) was estimated to be 32.10% (Table 2). When MARS was calculated, non-adherent participants scored lower MARS [16.76±5.02 vs. 23.04 ±3.24] than adherent respondents (P<0.001). Among adult participants, the prevalence of non-adherence was estimated to be 40.80% and non-adherent respondents scored lower MARS [16.54 ± 5.22 vs. 22.61 ± 3.54] than adherent ones (p<0.001). Regarding children, the prevalence of non-adherence (as reported by their mothers) was estimated to be 23.40% and non-adherent participants scored lower MARS [17.14±4.65 vs. 23.37± 2.94] than adherent ones (p<0.001). It is worth mentioning that there is a significant association between MARS and adherence level among all the participants, adults, and children (p<0.001).

**Table 2:** Non-adherence of participants to short-term antibiotics.

Participants	Not adherent	Adherent	P value^a^
All participants (n = 2000) n (%) MARS, mean±SD	642 (32.10) 16.76±5.02	1358 (67.90) 23.04 ±3.24	<0.001
Adults (n = 1000) n (%) MARS, mean±SD	408 (40.80) 16.54 ± 5.22	592 (59.20) 22.61 ± 3.54	<0.001
Children (n = 1000) n (%) MARS, mean±SD	234 (23.40) 17.14 ± 4.65	766 (76.60) 23.37± 2.94	<0.001

^a^ based on difference between MARS score

### Predictors of non-adherence to short-term antibiotic

The results of the univariate analysis are presented in S1 Table. The multivariate analysis of adults and children is presented in Table 3. Adult analysis showed the absence of comorbid condition to be associated with non-adherence (OR = 0.615, 95%CI = 0.444–0.853, p = 0.004). The analysis among children showed that higher number of doses per regimen was identified as a predictor for non-adherence to antibiotic (OR = 0.965, 95%CI = 0.950–0.981, p<0.001). When multivariate analysis was conducted for all the participants (adults and children), the results indicated that the mothers were 2.6 times more likely to be adherent in giving antibiotics to their children compared to adults taking antibiotics by their own (OR = 2.657, 1.844–3.827, p<0.001).

**Table 3:** Multivariate analysis of factors affecting short-term adherence.

**Factors** ^a^	**OR (95%CI)**	**P value**
	**Adults**	
Age	1.004 (0.993–1.014)	0.492
Presence of comorbidity Yes No	Ref 0.615 (0.444–0.853)	**0.004**
Number of doses per duration	0.994 (0.983–1.006)	0.306
	**Children**	
Age of child	0.966 (0.923–1.010)	0.126
Employment^b^ Unemployed Employed	Ref 0.771 (0.536–1.109)	0.161
Family income^b^ <500JD ≥500JD	Ref 0.821 (0.581–1.160)	0.263
Number of doses per regimen	0.965 (0.950–0.981)	**<0.001**

^a^ The multivariate analysis was conducted adjusting for variables with p<0.25 in the univariate analysis.

^b^ For mothers of children

### Reasons associated with non-adherence to short-term antibiotics

The participants reported different reasons associated to non-adherence: (i) patient related factor such as "unaware about consequences of stopping antibiotic" (77.55%), (ii) therapy related factor such as "taking medicines more than once a day is inconvenient" (72.85%) and “taking too many drugs (polypharmacy)” (60.60%), (iii) healthcare related factor such as “physicians did not explain the consequences of stopping antibiotics” (62.10%), and (iv) condition related factors such as “discontinue antibiotic as the condition improves” (63.15%). Detailed description is presented in Table 4.

**Table 4:** Reasons of non-adherence to antibiotics as reported by all participants.

Reasons	All participants	Adults	Children
	n (%)	n (%)	n (%)
**Patient related factors**			
Unaware about consequences of stopping antibiotic	1551 (77.55)	763 (76.30)	788 (78.80)
Lack of attention from family members	1508 (75.40)	636 (63.60)	872 (87.20)
Inadequate knowledge about illness	1374 (68.70)	688 (68.80)	686 (68.60)
Fear of adverse effects	1179 (58.95)	578 (57.80)	601 (60.10)
Too busy in study or work.	1173 (58.65)	641 (64.10)	496 (49.60)
Difficulty in buying antibiotic	560 (28.00)	239 (23.90)	321 (32.10)
**Therapy related factors**			
Taking antibiotics more than once a day is inconvenient	1457 (72.85)	767 (76.70)	690 (69.00)
Too many drug varieties (polypharmacy)	1212 (60.60)	617 (61.70)	595 (59.50)
Effect of drug wanes	1005 (50.25)	502 (50.20)	503 (50.30)
Drug prices	845 (42.25)	459 (45.90)	386 (38.60)
Difficult drug leaflet	566 (28.30)	281 (28.10)	285 (28.50)
**Healthcare related factors**			
Physicians do not explain the consequences of stopping antibiotics	1242 (62.10)	584 (58.40)	658 (65.80)
Lack of Pharmacists counseling	932 (46.60)	426 (42.60)	506 (50.60)
**Condition related factors**			
Discontinue antibiotic as the condition improves	1263 (63.15)	645 (64.50)	618 (61.80)

## Discussion

This study demonstrated that around 30% of the study population, including adults and children, was non-adherent to short-term antibiotic treatment in Jordan. Absence of chronic diseases and higher number of doses per regimen were associated with less adherence among adults and children respectively. Out of 4674 patients screened for participation in the current study, 2802 (60%) have used antibiotics within the last month. This indicates that antibiotics are commonly used among Jordanian population. Respiratory symptoms were the main reason for using antibiotics which is similar to the results of previous studies including Jordan [7, 18]. Since most of the respiratory infections are viral in nature, the antibiotics use was found to be high and inappropriate for such infections in Jordan [19].

More than forty percent of the adult participants in the current study were non-adherent to short-term antibiotic course which is higher than the rates reported in the literature. Llor et al. (2013) found that 25.2% of the participants were non-adherent based on self-reported survey in Spain [4]. In addition, Chan et al. (2012) found that 32.9% of the participants showed non-adherence towards antibiotics in China [8]. The definitions and reporting of non-adherence were different among the studies which have the potential to affect the prevalence rates; for example, Llor et al., measured adherence using medication bottles containing a microelectronic chip to register the date and time of opening of each bottle and non- adherence was defined as taking the medication less than 80% of the prescribed number of days/times [4]. In addition, Llor et al. (2013) and Chan et al. (2012) assessed adherence to treatment for specific indications while in our study antibiotics were used to treat several potential infections (respiratory, urinary tract, and gastrointestinal symptoms) without confirmed diagnoses. Furthermore, variation in the rates of adherence could be due to cultural differences that might affect the medication adherence behavior.

In the present study, the multivariate analysis revealed that children were three times more likely to be adherent than adults. This might be attributed to the fact that children are being taken care of by their mothers who are more serious and concerned about their children health compared to adults taking care of their own health. Parental beliefs, knowledge and attitudes play a major role in their children treatment and medication adherence [20–24]. Further studies evaluating the relationship between mothers’ knowledge and attitudes about antibiotics and the adherence among their children are recommended. In the current study, children showed a higher rate of adherence compared to other studies also measuring medication adherence among children with chronic diseases [25–27]. The presence of chronic conditions, chronic medication use, or/and polypharmacy has the potential to decrease the adherence in children overtime explaining the higher rate of adherence observed in this study.

The absence of comorbidities was associated with lower levels of adherence among the adult participants, this might be explained that patients with comorbid conditions become more cautious to take their antibiotics as per prescription to avoid more serious consequences on their health. These patients may perceive a higher burden of infections on their health. In addition, the current study showed that the number of doses per regimen had a significant effect on adherence among children; adherence decreases as the number of antibiotic doses increases. This is in line with the findings Llor et al. (2013) where adherence was inversely associated with the daily number of antibiotic doses and antibiotic duration [4]. Similarly, a meta-analysis also reported a higher compliance to antibiotic treatment with fewer daily doses [28].

Different reasons of non-adherence were reported in the literature such as healthcare, condition, therapy, and patient-related factors [2]. In the current study, the main patient-related factor was being unaware of the consequences of stopping antibiotic which is consistent with a previous study [13]. This proves the importance of healthcare professional’s role as main educator to patients about their illness, the proper use of medications, and the consequences of sub-optimal adherence. Appropriate patient education has the potential to improve treatment outcomes including adherence. Expectedly, inconvenience in taking antibiotics more than once daily or taking too many drugs (polypharmacy) were the main therapy-related factors in the present study. This is also consistent with previous studies reporting that multiple daily dosing reduced adherence among children and adults [29, 30]. This is also confirmed by our finding identifying higher number of doses per regimen as a predictor for non-adherence to antibiotic. Polypharmacy is a well-known factor associated with lower adherence rates [31, 32].

Regarding healthcare-related factors, “physicians did not explain the consequences of stopping antibiotics” was rated higher than of “lack of counseling by pharmacists” responses among the participants. This might be due to the fact that physicians do not have enough time for counseling and providing a detailed patients’ education as they are usually busy during the day with the high number of patients, diagnosis, prescriptions, etc. Pharmacists on the other hand, besides having the appropriate knowledge, they are usually more available and accessible to counsel and educate patients. Discontinuation of prescribed antibiotic once the condition improves was one of the main condition-related factors to non-adherence which remains consistent with previous studies [13, 33].

This study has some limitations. First, the potential for selection bias, due to the convenience sampling approach, the participants who were interested in the topic could have participated more than others. However, our participants were recruited from three main geographical areas of Jordan (north, south, middle) with a high response rate. Second, recall bias in answering questions related to antibiotic use (e.g., duration, dosing frequency …). To minimize the effect of this issue, the antibiotic use was determined within the last 30 days. Third, social desirability biases might have overestimated the actual rates of adherence. Fourth, subjective method for the assessment of adherence (based on respondents’ memory) was the only method used in the study. Future studies considering objective method (e.g., pill count) would be helpful to design appropriate intervention in Jordan.

## Conclusions

The present study revealed that more than half of participants were adherent to short-term antibiotics. Children’s mothers were more cognizant about antibiotic use and their children had a higher adherence rate when compared to adult participants. Additionally, adults without comorbid conditions and children with higher number of doses were less adherent to the antibiotic course. Views of participants about different non-adherence reasons highlighted the need of public education about the rational use of antibiotics. A chance for adherence improvement is present through the enforcement of resilient regulatory policies, and the activation of effective dormant pharmaceutical counseling.

## Supporting information

S1 Table Univariate analysis of factors affecting short-term adherence. (DOCX)
