# Supplementary material for: Prevalence and predictors of non-adherence to short-term antibiotics: A population-based survey
Source: PLoS One. 2022 May 19;17(5):e0268285. doi: 10.1371/journal.pone.0268285 (PMC9119442; doi:10.1371/journal.pone.0268285)
Supplement: S1 Table — (DOCX) [file pone.0268285.s001.docx]

**S1 Table. Univariate analysis of factors affecting short-term adherence.**

| **Factors^a^** | **Adults** | | | **Children** | | |
| --- | --- | --- | --- | --- | --- | --- |
|  | **Not Adherent**  **N=408** | **Adherent n=592** | **P value** | **Not Adherent**  **N=234** | **Adherent**  **N=766** | **P value** |
| Age^b^ | 34.59±13.97 | 36.67±13.78 | 0.020 | 5.54±3.12 | 5.10±3.32 | 0.070 |
| Gender   - Male - Female | 137 (33.58)  271 (66.42) | 187 (31.59)  405 (68.41) | 0.509 | 123 (52.56)  111 (47.44) | 430 (56.14)  336 (43.86) | 0.336 |
| Education level^c^   - School education^d^ - University education | 153 (37.50)  255 (62.50) | 241 (40.70)  351 (59.30) | 0.307 | 144 (61.54)  90 (38.46) | 477 (62.27)  289 (37.73) | 0.840 |
| Employment^c^   - Unemployed - Employed | 252 (61.76)  156 (38.24) | 379 (64.00)  213 (36.00) | 0.468 | 161 (68.80)  73 (31.20) | 565 (73.76)  201 (26.24) | 0.137 |
| Monthly income^c^   - <500JD - ≥500JD | 224 (54.90)  184 (45.10) | 305 (51.52)  287 (48.48) | 0.292 | 147 (62.82)  87 (37.18) | 520 (67.89)  246 (32.11) | 0.150 |
| Presence of comorbidity   - No - Yes | 310 (76.00)  98 (24.00) | 387 (65.37)  205 (34.63) | <0.001 | 204 (87.18)  30 (12.82 ) | 670 (87.47)  96 (12.53) | 0.908 |
| Source of payment for antibiotic^c^ Out of pocketInsurance  - Both | 102 (25.00)  115 (28.19)  191 (46.81) | 164 (27.70)  176 (29.73)  252 (42.57) | 0.398 | 36 (15.38)  81 (34.62)  117 (50.00) | 151 (19.71)  255 (33.29)  360 (47.00) | 0.328 |
| Number of antibiotics used in previous month One time  - ≥ two times | 278 (68.14)  130 (31.86) | 422 (71.28)  170 (28.72) | 0.286 | 179 (76.50)  55 (23.50) | 568 (74.15)  198 (25.85) | 0.470 |
| Number of doses per regimen^b^ | 16.66±10.57 | 16.37±11.54 | 0.686 | 16.20±7.83 | 13.30±9.41 | <0.001 |

^a^ All data expressed as n (%) of patients unless otherwise indicated.

^b^ Data described as mean ± standard deviation

^c^ For mothers of children

^d^ Primary and/or secondary school
